# Developing Ethical Practices for Public Health Research Data Sharing in South Africa: The Views and Experiences From a Diverse Sample of Research Stakeholders

**DOI:** 10.1177/1556264615592386

**Published:** 2015-07

**Authors:** Spencer G. Denny, Blessing Silaigwana, Douglas Wassenaar, Susan Bull, Michael Parker

**Affiliations:** 1 University of KwaZulu-Natal, Pietermaritzburg, South Africa; 2 University of Oxford, UK

**Keywords:** data sharing, data curation, research ethics, public funded research, South Africa, public health

## Abstract

The abundance of South African clinical and public health research data has the potential to unlock important and valuable future advances in biomedical science. Amid increasing calls for more effective sharing of individual-level data, commitment to promote access to research data is evident within South Africa’s public research sector, but national guidance and regulation are absent. This qualitative study examined the perceptions, experiences and concerns of 32 research stakeholders about data-sharing practices. There was consensus about the utility of data sharing in publicly funded health research. However, disparate views emerged about the possible harms and benefits of sharing data and how these should be weighed. The relative dearth of policies governing data-sharing practices needs to be addressed and a framework of support developed that incentivizes data-sharing practices for researchers that are both ethical and effective.

The abundance of research data that exists today has emormous potential to unlock future advances in science, a prospect that has been discussed by researchers and policy makers for almost three decades (Borgman, 2012). The sharing of health research data is of increasing interest, with many funders advocating for, or even requiring researchers to share data sets as a condition of funding to maximize their utility and value (Medical Research Council, 2014; National Institutes of Health, 2003, 2014; Walport & Brest, 2011; Wellcome Trust, 2009). However, data-sharing activities appear to be inconsistent and concentrated in a few select research fields (Borgman, 2012).

One area where data-sharing policy and practice is more developed is in genomics. Recent progress in data-sharing oversight for genomic data has made significant inroads worldwide, in terms of providing a consistent approach to sharing research data (National Human Genome Research Institute, 1998; Wellcome Trust, 2009). Policies mandating researchers to share data are becoming commonplace in biomedical research (Department of Health and Human Services, 2014; Wellcome Trust, 2009), especially for publicly funded research undertakings (Borgman, 2012). Similarly, several major biomedical journals now require authors of original research to provide a statement on data sharing in published articles, even making data sharing a condition of publication in some cases (Rathi et al., 2012).

In public health research, by contrast, this is not consistently the case. Despite its potential, the sharing of public health data remains unsupported by global guidelines or frameworks (van Panhuis et al., 2014), and there are often no research practices in place to support data sharing. In South Africa, for example, extensive data sets are generated from clinical and public health research, particularly HIV and TB studies. Yet, notwithstanding the widely accepted importance of data sharing (Bull, Roberts, & Parker, 2015, in this issue), primary individual-level data are usually not curated and shared with non-collaborating investigators, prior to publication of results (Manasa et al., 2014). The situation is not helped by the lack of resources and infrastructure and a myriad of other practical, legal, ethical and cultural barriers to data sharing (Manju & Buckley, 2012; Nelson, 2009; Pisani & AbouZahr, 2010; Tangcharoensathien, Boonperm & Jongudomsuk, 2010; Tenopir et al., 2011). Increasingly, however, moves are being made to address some of these problems. As a step toward facilitating data sharing, the Southern African Treatment Resistance Network (SATuRN) initiative recently established a database to curate and share individual-level data sets (e.g., HIV gene sequences, clinical, laboratory, treatment data) with other scientists in the region, and potentially to inform policies on HIV drug resistance in Southern Africa (Manasa et al., 2014). Furthermore, data-sharing policies recently developed by research institutions such as the Human Sciences Research Council (HSRC) illustrate the commitment of South Africa’s research enterprise to actively promote widespread data sharing (Lötter & van Zyl, 2015)—even if the current national regulatory framework has no specific guidance on data sharing (Department of Health, 2015).

Successful and appropriate sharing of public health data depends on the trust and confidence of those from whom such data are derived and relate to. However, very little is known about different stakeholders’ (policy makers, researchers, research ethics committees [RECs], and research participants) perceptions, views, and concerns regarding sharing of individual-level research data in South Africa, and in other low- and middle-income settings (Bull, Roberts, et al., 2015). Although there are some published studies evaluating the implications of sharing research data, predominately based on the views of North American stakeholders (McGuire et al., 2011; Trinidad et al., 2010), few studies document the views of stakeholders from low- to middle-income settings. The need to develop appropriate data governance and protective policies that are cognizant of local stakeholders’ perceptions and concerns is clear if South Africa is to keep pace with international research priorities and practices.

In this article, we report on the South African findings from an international exploratory qualitative study examining stakeholder experiences in five low- and middle-income countries (India, Thailand, Vietnam, South Africa, and Kenya) of, and views about, best practices in sharing individual-level data from clinical and public health research (Bull, Cheah, et al., 2015). As South Africa generates vast amounts of public health research data, it is hoped that South African perspectives on data sharing will be of interest both to higher and lower income contexts. The aim of this article is to provide an analysis of stakeholders’ views about data sharing in South Africa.

## Method

### Study Design

A multi-site case study design was used to collect qualitative data from a range of different research stakeholders across three study sites in South Africa (Bishop, 2010). Data from each research site generated both within-site patterns and cross-site syntheses of individuals’ perceptions, experiences and concerns about sharing data with external entities.

### Study Sites

Three large South African research organizations that routinely collect, curate, and share data within their respective fields and collaborative capacities, both locally and internationally, were approached as possible sample sites. Two primarily biomedical research organizations were contacted: one a low-risk institute that is primarily engaged in fundamental biomedical research and specimen collection (Site A), the second a large health research and clinical trials unit, focused on HIV, TB, and AIDS prevention research (Site B). The third site that we approached was a large research organization that conducts social scientific research for various governmental and external organizations (Site C; see Table 1).

**Table 1.** Sample Demographic Data.

	Site A	Site B	Site C
Nationality			
Black African	4	8	4
Indian/Asian	1	4	
White	7	1	3
Gender			
Female	6	11	5
Male	6	2	2
Age			
Average	39.3	48.6	44.9
Range	(24-65)	(32-72)	(32-59)
Duration of role			
Average years	2.3	7.6	5.5
Experience range	(3 months-6 years)	(5 years-10 years)	(3 years-12 years)

### Participant Selection

Participants selected for inclusion fell into two broad categories, namely, senior research stakeholders with some personal experience of data sharing, and junior researchers and community-level stakeholders who might be expected to be less aware of data-sharing practices (see Table 2). Purposive and snowball sampling methods were used to recruit participants. Thirty-two individuals were recruited in total. Of these, 25 participants were from Sites A and B and a further 7 from Site C.

**Table 2.** Stakeholder Sample by Study Site.

Stakeholders	Site A	Site B	Site C
Community research support team		2^a^	
Junior research staff	4^a^	6^a^	
Research managers	2^b^		2^c^
Senior researchers	5^b^	2^b^	3^c^
Policy and department managers		2^b^	1^d^
Executive members	1^b^	1^b^	1^d^
Total	12	13	7

*Note.* Data-collection method: ^a^Focus group; ^b^interview; ^c^teleconference group discussion; ^d^Skype group interview.

### Data Collection

Qualitative data-collection tools were initially generated in collaboration with the international study partners in Kenya, India, Thailand, Vietnam, and the United Kingdom, and then tailored specifically to the local research context (Bull, Cheah, et al., 2015). These included an interview topic guide, to solicit responses from senior research stakeholders (available upon request) and focus group discussion materials, designed to assist in exploring a range of views about data sharing in a more structured way, using two specific vignettes. The first aimed to explore issues around sharing HIV/TB clinical trial data, which involved a South African case study and prompted focus group participants to consider the potential harms and benefits of re-using clinical data. The second vignette discussed the re-use of community health survey data by various kinds of research stakeholders and their potential benefits for the host community (available upon request).

Research with participants from Biomedical Sites A and B was conducted face-to-face, at participants’ convenience. Interviews with participants from Site C were conducted via Skype and telephone call on two separate occasions.

Sociodemographic information collected about study participants included age, sex, nationality, and primary employer, as well as professional characteristics, including level of education/qualification, academic background/area of study, current job title, and the duration of participants’ current position. The data-collection phase occurred over 5 months between May and September 2014.

In total, 20 senior research stakeholders were accessed for face-to-face and group interviews. Two focus groups were convened at Sites A and B with 12 junior and community research staff (See Tables 1 and 2).

### Coding Frame

Two authors (S.D. and B.S.) independently read each transcript and checked its accuracy against audio recordings. Transcripts were then coded using NVivo 10 software (QSR International, 2012), which was also used to manage subsequent data analysis. The coding process was initially guided by a “top down” coding frame that was developed collaboratively among team members involved in this larger multi-site study (Bull, Roberts & Parker, 2015). The majority of the codes were maintained throughout the coding process, with descriptive sub-codes relevant to this specific data set added where appropriate. The “top down” coding frame ensured a systematic method of organizing data extracts into meaningful groups throughout the co-coding process.

### Data Analysis

Coded data were analyzed using a thematic framework approach (Ritchie & Spencer, 2002). Key features of this approach include a grounded analysis of data, a dynamic and reflexive adaptation to change throughout the analytic process, and a systematic treatment of all units of data, allowing between- and within-case comparisons to be made across entire data sets. Thematic references were generated inductively by identifying emergent issues that were salient across the codes (Ritchie & Spencer, 2002). Analytic treatment of the data was reliant on the coders’ judgments about the purport of remarks made by respondents in response to the research question or prompt. Emergent themes were discussed between the coders at various times as well as with the research members from the other teams in the international study. This process of analysis involved tabulating or “charting” coded data to appropriate themes (Ritchie & Spencer, 2002). In this way, themes emerged by identifying patterns of shared meaning across the data set (Braun & Clarke, 2006).

### Data Management

Collected data including audio recordings and research participants’ demographic information were stored using coded identifiers. Information for re-linking individual identifiers to the raw data was securely stored on password-protected computers accessible only to the South African team. Research outputs such as transcripts, NVivo coding summaries, and thematic framework analysis charts were securely backed-up on an international server (SharePoint) hosted and managed by the lead research team at the Ethox Centre, University of Oxford. The SharePoint site provided a secure collaborative platform among the research partners to share data.

### Data-Sharing Plan

Permission to share individual-level data from this qualitative ethics project with collaborating teams was obtained during the consent process. Shared data consisted of de-identified transcripts, demographic data, NVivo outputs (coding summaries and emergent themes), analysis charts, and manuscript drafts. Data from the study are available, please contact the corresponding author for details.

### Ethical Considerations

The study received ethics approval from the Humanities and Social Sciences REC (HSSREC) at the University of KwaZulu-Natal (Approval Number HSS/1222/013), as well as from the Oxford Tropical REC (OxTREC–Approval Number 1051-13). All participants received a monetary token (equivalent to US$9) as compensation for their time, in accordance with South African research ethics guidance and as approved by the HSSREC. Written permissions were obtained from the CEOs of each organization to invite their staff to participate in this study. Written informed consent was given by all who participated in this study. A categorical (or tiered) consenting method was used, whereby study participants were asked to choose from a list of three “conditions” of participation. These conditions were (a) consent to participant in research, (b) consent to be tape recorded, and (c) consent to share individual-level data. Consent was obtained for each condition of participation from all participants.

## Results

In the following sections, we report on our analysis of the data collected in interviews with senior and junior researchers, sampled from three major South African research organizations, regarding their attitudes toward and experiences with sharing individual-level research data beyond their immediate team and established collaborative networks. Although most senior stakeholders at each of the research sites had some knowledge of what data sharing is, not all of the participants were aware of specific data-sharing policies, procedures, or issues that could arise when data are shared. For this reason, participants were provided with an introductory distinction between data shared in collaboration with partners, data shared through publication of results, and data collections shared on request by an external entity. It was explained that this study examined the latter form of data sharing.

### Stakeholders’ Predisposition to Data Sharing

In general, participants described data-sharing practices as either “*ad hoc*” decisions (post study) and informal practices of exchange between colleagues and interested persons (i.e., foreign exchange students), or as formal procedures, enforced by institutional policy in the form of contractual agreements between the principal investigator, their home institution, and the funding body. A clear example of this was the operationalization of one site’s data-sharing policy. This policy was developed about 3 years ago for the re-use of data collected on behalf of local and international clients. The policy was aimed to align with the organization’s founding legislation. One of this site’s annual performance indicators requires reporting to its core funders on progress made on the implementation of the institution’s data-sharing policy.

Interviews with respondents from Site C tended to focus on the organization’s data-curation activities, which included data-management processes, the review of external requests for access to available data, negotiation for the re-use of donor-funded research data collected by the site’s main research programs, and the implementation of data-sharing plans across research departments. Data sharing within this setting was generally described as being actively encouraged. However, Site C participants also identified several obstacles associated with institutional data-sharing practice. For instance, at the time of implementing the data-sharing policy as a management activity, one senior manager notes that “. . . people didn’t really think about data sharing, [as] I think they were less understanding of the benefits . . .” Adding that in her experience, a key issue that was faced when trying to introduce the new open data policy was the “. . . challenges, in terms of [changing] people’s attitudes and [growing] a sharing culture . . .” within the organization (Manager, female, Site C).

In contrast, interviews conducted at both of the biomedical sites tended to focus on researchers’ experiences of policy frameworks on specimen sharing and sample storage, namely, the use of Materials Transfer Agreements (MTAs) and memoranda of understanding (MOUs) when sending various kinds of research outputs to external entities. Sharing research data among these participants was frequently seen as an extension of collaboration between researchers, rather than as official protocol.

The perceptions, views, and concerns that respondents had about making their data accessible to a broader community of researchers and other potential interested users are reported below.

### Why Share Research Data?

The primary global value of data sharing, described by many of the senior researchers, was seen to be its potential to move the field of science forward by opening up new avenues of science and by closing knowledge gaps through collaborative communication between different research programs. This, in turn, was seen to have the potential to complement and enhance the responsiveness of research to public health needs and validating scientific outputs over time, reducing the duplication of scientific effort, and minimizing research costs. Participants’ benevolent attitudes toward advancing scientific knowledge for the greater good and providing public benefits to local communities were contrasted with the competitive value of research data, in terms of advancing participants’ careers.

### Disadvantages of Data Sharing

For most senior researchers, data were described as the lifeblood of their work. Data were inextricably connected to their research outputs, which are linked to publications, which in turn are linked to future funding. The need to protect data for its publication value was identified by several participants to be a key deterrent to releasing data, as some researchers worried that data would lose their value once placed in the public domain. The potential for shared data to be misused, misunderstood and produce false conclusions that threaten the integrity of the primary research was also highlighted. The problem of free riders was perceived to “. . . compromise people’s ability to be able to participate in advancing . . . science” (Scientist, female, Site B), in situations where, for example, “I spend my life collecting data and you spend your life analysing data and you will be the one getting the credit [for my work]” (Manager, female, Site B). Some senior stakeholders felt anxious that data sharing may resemble “neo-colonialist behaviour . . . where the raw materials are taken out of the country and the beneficiation happens outside and South Africa is the poorer for it” (Manager, male, Site A).

### Attitudes Toward Sharing Data

Although none of the stakeholders in this study categorically objected to sharing de-identified data for academic and public health purposes, there was some disagreement about the extent to which research data should be shared beyond this. For example, some senior respondents from the Sites A and B felt that not all data are of equal value and data should only be shared in certain circumstances. Reasons for sharing included pressures from government or funders, or an overwhelming public health interest in sharing data to prevent or minimize a disaster. Others in this group said that the extent to which data are shared would largely depend on the nature of the research question and whether the data could answer the question. Some researchers, by contrast, asserted that all data should be shared, all of the time, suggesting that “the more the data is made available the more likely it is to lead to scientific impact” (Researcher 1, male, Site A). Participants also had divergent views about the degree to which data should be made freely accessible, with some suggesting the need to restrict and gate-keep the re-use of data, whereas others believed that researchers should be required to relinquish control over data collections once curated, or on release, unless under embargo.

Decisions about releasing data were discussed in relation to informed consent and researchers’ responsibilities to respect research participants’ confidentiality when sharing data.

### Informing Research Participants About Data Sharing

Among the community research support members and junior research staff at Site B, agreeing to share data for future research was seen as an altruistic act, but one that needs to be respectful of people’s rights. These included participants’ right to know the following:

- the intended purpose of future research
- the potential risks and benefits of future research done using shared data
- how the researcher might benefit from sharing his or her data
- how the host community might be appropriately acknowledged for its contribution to scientific knowledge

This group of participants felt very strongly that “if [the re-use of data] is anything besides what I initially consented to then I need to know and [be] informed. It’s my right to know” (Counselor 8, female, Site B). The particular use of data for which consent was obtained must be respected in future research. It was the responsibility of the researcher to brief participants in appropriate detail about data-sharing plans, and in terms that are meaningful and actively negotiated during the consent process. For example, a respondent would feel cheated “if they are accessing my information [for new research] just because I was participating in a study at this research organisation, then it is stealing” (Community Advisory Board Member 1, female, Site B).

In recognition of the then current South African Research Ethics Guidance (Department of Health, 2004), a senior scientist from Site C explained that participants should give informed consent and should also consent to what happens to their data after the study, adding that it is the responsibility of researchers to

. . . ask the participants on the basis of the information sheet to consent to the study being done, [and for] the data to be anonymised, captured and shared . . . for a specific purpose. So you cannot say for instance, draw blood to study . . . the prevalence of blood groups in South Africa and then suddenly do HIV/AIDS research on that. (Researcher, male, Site C)

The problem with giving a more focused statement on data sharing during consent was that

. . . we do not know what the value of that data will be for different purposes and . . . from an administrative point of view to guarantee the use of data for specific purposes, is not practically possible. (Manager, female, Site C)

This becomes more complex when dealing with several hundred data sets that would require a significant amount of “. . . person-power to audit what happens once participants sign a consent form [to share data]” (Manager, female, Site C). In addition, several respondents feared that additional information about how data would be shared during consent might discourage research participation. They suggested instead that consent focused on “the main issues of . . . ethical research” (Manager, male, Site A) that relate to participants’ safety and the commitment required by their participation. However, the general consensus among senior researchers was that a clear but broad statement on the potential re-use of participants’ data by other people for future research was more appropriate than specific details regarding how, where, and by whom it would be accessed.

This conflict between the ability of research participants to control how their data are re-used and the uncertainty of future research endeavors emerged as an ethical dilemma when respondents were asked to consider specific approaches to seeking consent. They expressed differing views about the best ways to protect the rights of the participants and the aims of the research. This is discussed further below.

### Preferred Methods of Consent

In general, senior participants from all three sites preferred a broad approach to consent, in which consent was obtained for future research related to the primary research area. In this way, senior interview participants felt permitted to conduct future research on existing data or even share data with others when it was within the original field of study, without an explicit indication of data-sharing plans. For example, a senior researcher from Site A explained that

. . . in my consent forms, I think what they [the participants] have agreed to is that their samples will be used for people . . . to do immunology on their samples . . . we just tell them we are going to do immunology and they have agreed for us to do the immunology. (Researcher, female, Site A)

The following advantages of broad consent were outlined:

. . . it’s easier to get genuine informed consent if you have a single clear thing that you are consenting for. (Researcher, 1, male, Site A)

. . . blanket approval [helps to] facilitate research [which] in this environment it’s often very difficult to even trace patients to get the informed consent . . . to do something new. (Researcher, female, Site A)

Although many respondents advocated the use of this method, ethical unease was expressed about conflicting assumptions about the scope of informed consent and the merits of this approach. An example was when consent to share data was assumed to be implicit in research participation rather than delineated in a meaningful way. One respondent noted that

. . . one of the drawbacks [to broad consent] is that . . . the patient or participant is now less in-the-know . . . they do not really know what will happen to their data so they are less informed to make a truly informed decision. (Manager, male, Site A)

Competing views about the merits of providing more detailed information about sharing individual-level research data emerged in relation to interviewees’ desire to exercise some control over data they had collected when sharing them with external entities. At the same time, they expected there to be only minimal restrictions on the uses they could make of data when granted permission to perform secondary analysis from an external source.

### Data Management

The process of curating information generated by research in a retrievable and auditable manner raised several views about the need to protect data from misuse and the commitment by researchers to accurately preserve data for future re-use. The ethical duty of researchers was described by many interviewees as the provision of accurate data records to nurture professional integrity through transparency of practice and to avoid unauthorized future use of research data. Almost all participants agreed on the importance of having properly specified metadata in this regard. Efforts to ensure secure and ethical management of research data included being able to provide carefully stipulated data preservation plans, in which investigators were required to indicate the type of data generated, its potential scientific value, and options for data access. In Site C’s experience, data-sharing plans were built into research protocols submitted for ethics review in “. . . an attempt to make [researchers] aware of data sharing from the outset so that they also consider [its implications in] the informed consent form” (Manager, female, Site C). This also required researchers to specify a time frame within which to release data for secondary access. Generally, it was felt that sufficient time must be provided for the publication of the primary analysis. There was some agreement among respondents that following data release, there should be limited constraints or restrictions on the re-use of curated data. In addition, they argued that overly bureaucratic approaches to data sharing should be avoided, lest they lead to reduced academic agency to freely pursue secondary analyses of interest.

A data-management system facilitating data re-use must take into account the “. . . ethical obligation for the researcher to share data at the end” (Researcher 2, male, Site A) of a study by protecting research participants’ confidentiality and the responsibility of the end user to respectfully engage with shared data.

### The End User of Shared Data

Two salient types of response regarding the role of the end user were given by our study groups. The first concerned the recipient of shared data, and the second referred to the validity of data re-use requests. Of particular concern was the potential threat of research misuse. Community and junior research support staff referred to the possibility of harm created by imprecise or stereotyped reporting, for instance “. . . like, if they are showing Africa they show a child with a fly on a nose all the time . . . the information is being exaggerated” (Counselor 2, female, Site B). An additional potential harm related to perceived diminishing prospects of benefits of secondary research for the community from which the data came. This was exacerbated by potential geographical detachment between the data source and data end users.

It is a good thing that research being done should start at that community. It is not a good thing for the research to be conducted [by] students from outside South Africa [who] will use data to develop communities in their own countries [and] the community from where the study was conducted does not get anything. If there is research in a certain community it should be carried out by people in that community instead of people outside that community. (Counselor 3, female, Site B)

This raises the issue of whether secondary data users should be asked to consider potential benefits to original participants when requesting data. Similarly, when asked to comment on access to community health data by foreign students for secondary analysis, community and junior research support staff from a rural Site B research clinic felt that additional regulations to protect the community’s interests should be applied to non-local data-access requests. These would aim to promote reciprocity that “. . . acknowledge[s] the contributions and good partnership and working together with the community” (Counselor 3, female, Site B).

For senior researchers, the primary concern was ensuring the scientific validity of secondary research. It was imperative for the end user to be able to exhibit, on request, that they have “. . . engaged with what they want to use the data for on a conceptual level” (Manager, female, Site C), which obviously means reviewing metadata catalogues and other documentation on the data nuances. The competence of the end user to re-use data appropriately would be assessed by means of either a “concept sheet” referred to by Site A and B participants, or an online submission of an application to the curation center at Site C. Both application methods require an outline of the research purpose, its aims and objectives, proposed methodology with specific reference to how the data would be used, benefits to be gained from sharing, and some effort to show “. . . how they would inform the original community that their data is being used in other research and when and how it could potentially bring back those findings” (Researcher, male, Site C). This last point introduces the need to provide feedback on reports of research uses, which is explored later in this article.

None of our participants saw private-sector entities as appropriate recipients of shared data. Instead, most respondents emphasized the need to prohibit profit-driven secondary research done using their data. They argued that data from largely publicly funded organizations should not be shared for for-profit purposes. Public-sector research was perceived as being guided by academic inquiry and motivated toward public benefit, placing importance on peer-reviewed publications and scientific replication. Speaking from past experience, a Site A member noted that “. . . the only obligation on the part of the recipient was not to commercialise the product” (Researcher 3, male, Site A), largely because participants who would share data do so freely. The idea of sharing data for profit seemed to invoke in many of our interviewees a sense of injustice in terms of the balance of rewards between those who contribute toward sharing data and those who stand to gain from its re-use. However, as one respondent suggested, should the re-use of data generate something of commercial value, then

. . . we would prefer to . . . make that a publicly accessible intellectual property or donate it . . . to the government of South Africa [and] would prefer that the beneficiaries are South African locals as opposed to some commercial entity. (Manager, male, Site A)

Comments about the accountability of the end user, in general, tended to focus on enforcing the rights of the primary researcher, which included being informed of outcomes generated by the end user and being acknowledged as the original data creators in subsequent publications or outputs. Although these requirements encourage fair practice in data re-use, some senior participants felt that in post data release, “there [are] very few controls that you can effect” over the future use of data in practice, “I mean it all has to do with trust” (Manager, male, Site C). For instance, a senior manager from Site C explained that compliance with the End User License, which requires that the data-curation center be informed of all outputs derived from shared data collections, has been quite low and difficult to enforce because “there is actually no real way that we can track the use of the datasets” once in the possession of the end user (Manager, female, Site C). Despite the fact that access to online data collections is governed by a time-dependent expiration of the end user’s subscription to the data provider’s online service, the downloaded content may remain in the end user’s possession indefinitely.

### Data Retention

The ease of transnational transmission of digital research data emerged as a significant perceived threat to researchers and research participants alike, leading to the need for constraints when exporting data and research materials outside the host country and the re-use of data for commercial exploitation. The continued use of data was understood by most respondents to be governed by two official documents: first, in relation to what the informed consent form specified will be done with individual research data and, second, in terms of the contractual obligation to guard privileged information on behalf of the research funder or sponsors. However, the issue of data ownership pending its release to a broader scientific community was regarded as complex; several respondents said that the right to retain data is dependent on

. . . whether the informed consent form [authorizes] the transfer of those rights from the participant to the investigator or the sponsor—if they [the participant] have not . . . agreed to transfer their rights of the data [then] neither the sponsor, nor the investigator, [nor] the collaborator outside of the institution can actually say that they own the data. (Manager, male, Site B)

This suggests, then, that researchers should not be viewed as owning the data but rather as having custodial responsibilities and rights over it. If they were to transfer data to an external collaborator for re-use, the recipient is then merely the possessor of the data, with only the right to possession. So although the researcher can assume some control of the data, on the understanding that this brings with it certain responsibilities, “the owner may [indeed] be the research participant” (Manager, male, Site B). Most other respondents thought that the research funder reserved the right to share data because he or she possessed the intellectual property rights for that data.

In light of this last point, funders were viewed as obligated to provide guiding principles and parameters for data sharing because they were usually responsible for setting the research agenda. As funders

decided that this study is worth doing I would hope that [they] who fund studies with some big picture in mind . . . would know where else this data can be used to advance the big picture [of research]. (Manager, male, Site B)

Attitudes toward sharing data at a community level reflected the need for policy to address not only the purpose for which data are re-used but also the nature of research being carried out in such areas. This suggests that the value of shared data to contribute to new knowledge should be measured in terms of the actualization of public benefit for these communities. Similarly, a researcher from Site B expressed reservations about sharing data with developed country partners because, by doing so, local opportunities to develop research infrastructure and personnel might be compromised by handing over research data to be analyzed by entities that already possess the technological and technical capacity to generate findings rapidly. Meaningful interaction and scientific involvement between stakeholders should aim to ensure that data sharing done within this context does not resemble neo-colonial dynamics and become a “mining process” between “. . . investigators from the North needing access to samples [data] in the South” (Manager, male, Site B). It was suggested that data-sharing processes needed

. . . to recognise that there are infrastructure and systemic imbalances between investigators of the North and the South . . . in terms of where the funding comes from and where . . . the research infrastructure is and so you may find instances where developing world scientists actually do the work . . . and where somebody else who may have access to the dataset, let’s say Oxford or Harvard, who can do that in ten days ends up then poaching the data and publishing in the field with data that’s not actually theirs, because it is put in the public domain. (Manager, male, Site B)

### A Benefit Sharing Component

A widely held view among our interviewees was that data-sharing agreements should include clauses requiring the preparation of metadata and dissemination of results with the view to public health implementation. This would require the researched communities to be involved. “You do not do research on communities you do it with them” (Researcher, male, Site C). Research was seen as a shared task between stakeholders, and by sharing individual data, “. . . you are also participating in that person’s study” at a later stage (Researcher, female, Site A). It could be said that “. . . making data available [for re-use] actually demonstrates respect for the respondents, in that you care about what they’re saying, it’s not just something that you use and discard” (Manager, female, Site C). Budgeting for the dissemination of research outputs of shared data was seen as necessary, where appropriate, by some senior researchers in our sample.

Some said that the implementation of data-sharing practice should not be at the expense of the community. For example, a senior manager from Site B recommended that

. . . there has to be a benefit sharing component that’s in the data sharing process and the benefit sharing has to be . . . done in a critical way where there is not just benefit for the investigator who is now going to have a patent and generating billions versus the community who’s still living in poverty. (Manager, male, Site B)

Typically, in primary research, concerns about risks and benefits to research participants are considered in two ways: (a) minimization of foreseeable harms and compensation for any inconvenience incurred by the study and (b) the maximization of benefits from the knowledge derived from the study. Researchers emphasized the need for research participants to be made aware of the protected nature of research and the often indirect benefit from the study. The majority of our sample strongly advocated for disseminating feedback of research results to the participant community to acknowledge research participants and their contribution to science. There are, however, several challenges to disseminating community feedback, which senior participants identified as follows:

- The difference in openness and receptiveness of different communities toward receiving feedback
- The careful steps researchers have to take to de-identify research participants
- Lack of funds to report back to individual participants

Several stakeholders felt that providing feedback to participants was a critical aspect of good data-sharing practice, and that appropriate plans for dissemination of feedback should be required by RECs as a condition of approval. As participants from Site C explained, such costs would fall on the organization, which provides data free of charge to end users. An executive stakeholder added that “it costs money to curate [data]” and that the costs of data curation are not covered by a typical research grant (Manager, female, Site C).

## Discussion

To the best of our knowledge, this is the first study in South Africa to explore stakeholders’ perspectives regarding sharing of individual-level data from clinical and public health research outside existing research collaborations. The findings reported here illustrate that, in general, research stakeholders are supportive of data sharing. Although we do not aim to provide definitive analyses, it was evident that local researchers support data sharing for multiple reasons, which are similar to those in the wider international literature (Bull, Roberts & Parker, 2015). However, community members and junior research support staff mostly cited altruistic reasons and prospects of health benefits—and monetary rewards (in some instances)—for their willingness to share data with researchers. According to Zarin (2013), honoring the altruism of research participants is an important principle of best practice in data sharing.

Nevertheless, despite general support, our study also showed that stakeholders’ concerns about confidentiality, data misuse through commercial exploitation, and fears that reduced data exclusivity will lead to lost opportunities for further research were prominent reasons against data sharing. Some researchers’ views suggest that benefits for data sharing outweigh potential harms. Consequently, arguments should move beyond the question of whether to share data or not (Chalmers et al., 2014) to how to share in ways that best minimize potential harms and respect participants’ reasonable expectations. Some commentators have identified the need to ensure that benefits outweigh potential harms as a principle to inform best practices in ethical data sharing (Vickers, 2006). Our findings are consistent with a substantial amount of literature from developed countries detailing the advantages and potential harms of data sharing—and general support for data sharing (Bull, Cheah, et al., 2015).

Our findings confirm that informed consent is seen as a key concern when sharing research participants’ data and medical records. Of importance were strong sentiments from community and junior research support staff about the need for more specific consent for research purposes differing from those for which original consent was obtained. In contrast, most senior researchers strongly favored broad consent, primarily for practical administrative reasons, such as mitigating challenges of tracking participants to obtain specific consent for unknown future research. Such evidently divergent views speak volumes about the recurrent debates regarding the appropriate consent process for data sharing (McGuire et al., 2011; Wallace, 2013).

Consent was not the only important theme in our findings. Researchers, for instance, regularly raised concerns about rights and ownership of data. Commentators have advocated the need to ensure the protection of rights and responsibilities of investigators generating data while recognizing the rights of data accessors (Sankor & IJsselmuiden, 2011). This includes ensuring that investigators are duly acknowledged in publications for their role in producing primary data sets (Pisani & AbouZahr, 2010; Rathi et al., 2012; Smith et al., 2014) and authorship rights (Pearce & Smith, 2011; Savage & Vickers 2009). However, community and junior research support staff (and some researchers, of course) reiterated their desire for transparency and accountability as well as equitable benefit sharing and local capacity development. These findings are consistent with priorities identified in both developed (Mello et al., 2013; Walport & Brest, 2011) and developing countries (Sankor & IJsselmuiden, 2011; Tangcharoensathien et al., 2010).

Our findings have important policy implications and together with those of our partners in Thailand, India, Vietnam, and Kenya have the potential to inform future data-sharing policies in South Africa and other low- and middle-income settings. The current regulatory framework in South Africa is silent on data sharing. Thus, we hope the results of our pilot study will pave the way for further discussions on the development and implementation of data-sharing policies. Policies must address stakeholders’ concerns in a harmonized way to ensure ethical data-sharing endeavors locally and internationally.

In addition, our data suggest that RECs can potentially play a crucial role in data sharing by safeguarding research participants’ rights and welfare. We observed that most researchers alluded to ethics oversight by RECs as being an inherent and compulsory process in data sharing. However, one senior researcher cautioned against overly procedural approaches to data sharing, in particular from RECs, citing fears that this may obscure and hinder the “bigger picture” of data sharing. Conversely, community members did not express views about the role of RECs in data sharing, and it was unclear whether community members were aware of such committees and their potential role in data sharing. We did not sample REC members, and there were differing views about whether RECs should form part of data access committees or governance mechanisms for data sharing. Further studies with REC members may be useful in exploring their views of, attitudes to, and perceived responsibilities in data sharing.

This study is not without limitations. First, we only sampled a relatively small number of stakeholders. Although our findings hopefully contribute to debate, it is important to acknowledge that results may not be generalizable to South Africa as a whole or to other low- and middle-income countries without further research. Also, our sample consisted of only two community members. Considering that they are such an important group of stakeholders, our findings do not reflect research participants’ voices. Another limitation is that we used convenience sampling to identify interviewees. Research stakeholders with greater experience of data sharing may have been omitted from our sample. Last but not least, time constraints precluded follow-up interviews with stakeholders. This would have been valuable to provide more insights into some emerging themes lacking clarity such as best practices in data-access procedures and governance.

In summary, our data demonstrate that both researchers and community members support the idea of data sharing in general. However, understandably, there are practical and cultural barriers to sharing data. Lack of infrastructure and resources is one of the many barriers, given the relative underdevelopment of South Africa (Manasa et al., 2014). Against that backdrop, funding opportunities or subsidized costs from funders to establish data-curation services and facilitate data-sharing processes should be made available to research institutions to develop and maintain curation infrastructure and support related activities. Participant feedback and data-curation costs could be made a standard budget line item in all research grants and be required as a condition of ethics approval.

## Best Practices

Funder-directed support in this area is recommended to assist local research stakeholders to develop appropriate data-curation procedures. Additional areas of support include the following:

- Access to resources based on relevant policy documents, data-sharing procedures, and relevant publications about data-sharing issues
- Documentation on general data-sharing guidelines for funders, researchers, RECs, and host communities
- Access to data directories

To assist in providing support in these areas, we have developed an online resource about ethics and best practices in sharing individual-level data, which is available at https://bioethicsresearchreview.tghn.org/research-data-sharing/.

## Research Agenda

There is need for further research with a broader range of experienced researchers, research participants, and RECs to address the themes identified in our study. Current South African legislation has yet to address the potential implications of global collaborative health research data sharing. Individual research organizations are, however, developing their own policies and practices in the interim. Given the novelty of this practice, we recommend further policy research in this area focused on ways to inform national ethical research guidance.

## Educational Implications

The implementation of data-sharing considerations in the ethical review of health research is not yet enforced as standard practice by many RECs in our setting. We believe that building data-sharing plans into research protocols submitted for either research grant review or ethical review would serve to strengthen ethical oversight and practices in this context. Furthermore, the development of resources should be prioritized to support the decisions made by primary researchers, as well as to inform community advisory boards about data-sharing issues so that they can better represent the interests of their communities.
